# Report on Maternal Anxiety 16 Months After the Great East Japan Earthquake Disaster: Anxiety Over Radioactivity

**DOI:** 10.5539/gjhs.v6n6p1

**Published:** 2014-06-24

**Authors:** Hatsumi Yoshii, Hidemitsu Saito, Saya Kikuchi, Takashi Ueno, Kineko Sato

**Affiliations:** 1 Health Sciences, Tohoku University, Graduate school of Medicine, Miyagi, Japan; 2 Department of Psychiatry, Tohoku University Hospital, Miyagi, Japan; 3 Division of Clinical Psychology, Graduate School of Education, Tohoku University, Miyagi, Japan

**Keywords:** child care, disaster area, earthquake, maternal anxiety, radioactivity

## Abstract

The Great East Japan Earthquake occurred on March 11, 2011. The tsunami caused extensive damage to the Fukushima Daiichi Nuclear Power Plant, resulting in a level 7 nuclear accident. Among those affected by this combined disaster were many pregnant and parturient women. Sixteen months after the earthquake, we conducted a questionnaire survey on anxiety among 259 women who gave birth around the time of the earthquake in Miyagi Prefecture, one of the affected areas. Participants reported 12 categories of anxiety, including anxiety over radioactivity. This study aimed to determine anxiety over radioactivity among this specific population and to record measures for future study. Anxiety over radiation was classified into seven subcategories: food safety, outdoor safety, effects on the fetuses of pregnant women, effects on children, radiation exposure, economic problems, and distrust of information disclosed. This study confirmed that concrete types of anxiety over radiation were keenly felt by mothers who had experienced the disaster who were currently raising children. The findings suggest the need to provide accurate information to these mothers, who are otherwise inundated with miscellaneous confusing information.

## 1. Introduction

The Great East Japan Earthquake occurred at 2:46 p.m. on March 11, 2011 (Yasumura et al., 2012). A tsunami hit the northern part of the main island of Honshu, covering more than 800 km of the Pacific coastline and claiming nearly 20 000 lives (Sato, Kobayashi, Ishibashi, Ueda, & Suzuki, 2013). For several months, residents of coastal areas had to live with major damage to electric power, water, and city gas supplies as well as lack of access to communication services (e.g., failure of phone lines, mobile phones, and internet connections) and shortages of food, fuel, and other essentials of life (Omama et al., 2011). The tsunami also caused extensive damage to the Fukushima Daiichi Nuclear Power Plant, resulting in a level 7 nuclear accident (Sugiura et al., 2013). The Japanese government released a statement comparing Fukushima with Chernobyl (Alder-Collins et al., 2013). The Fukushima Daiichi Nuclear Power Plant disaster caused environmental contamination by releasing fission products broadly in eastern Japan. The disaster also attracted a great deal of attention and caused extensive anxiety over the health risks of low-dose rate and low-dose radiation exposure resulting from radioactivity in residential environments (Ogiso et al., 2012).

Sixteen months after the earthquake, we conducted a questionnaire survey on anxiety among 259 women who gave birth around the time of the earthquake in Miyagi Prefecture, one of the affected areas. A beneficial way to reduce maternal anxiety would be to develop systems that provide continuous support for children’s mental health care needs, psychological guidance, community support for maternal empowerment, outreach for individual support, and professional consultation for mothers who have high anxiety about radioactivity. There is one large difference between past earthquake disasters and the Fukushima Daiichi Nuclear Power Plant disaster: The earthquake and tsunami disaster was also combined with a man-made radiation accident. In this paper, we consider detailed findings from a survey on radiation anxiety and discuss how the relevant organizations and bodies have dealt with anxiety, with a special emphasis on the keyword “radiation accident.”

## 2. Methods

### 2.1 Subjects

Study participants were mothers who had delivered a child less than one month before the Great East Japan Earthquake and pregnant women who had received a copy of the handbook issued by Japanese municipal governments, in accordance with Article 16 of the Maternal and Child Health Law, for pregnant women who report their pregnancy to a municipal office (“Maternal and Child Health Handbook”). We contacted 26 medical institutions in Miyagi Prefecture and asked them to explain the purpose of this study to their patients who gave birth during February–September 2011. Among the 886 women who initially agreed to participate, the final sample comprised 259 women who returned completed questionnaires. We administered the survey in July 2012, and the details of this survey have been previously described elsewhere (Yoshii et al., 2014).

### 2.2 Ethical Considerations

Approval to conduct this research was obtained from the Graduate School of Medicine, Tohoku University and hospitals and government offices in Tohoku, Japan that agreed to participate after receiving an explanation of the study. We informed the participants that the data collected would be used only for research and that they could withdraw from the study at any time. We explained that their participation would be kept confidential. Before starting the investigation, we obtained signed informed consent forms from all participants (see Yoshii et al., 2014 for a detailed description of this process).

### 2.3 Questionnaire Survey

The survey questionnaire included open-ended questions asking participants to describe their anxieties. After collecting basic demographic information, the questionnaire began with the general question, “What is causing you anxiety?” Participants could freely express their responses to this question, as has previously been described in detail (Yoshii et al., 2014).

### 2.4 Analytical Methods

We chose a qualitative study design (Howitt et al., 2010) and conducted the analysis through an iterative process. We used ATLAS.ti, Version 7.0 (Scientific Software Development GmbH, Berlin, Germany) for the data analysis. The first step of the data analysis involved repeated reading of the questionnaire responses to facilitate familiarity with the data, allowing for the beginnings of an interpretative process. To enhance the credibility of the analytical process, the co-authors analyzed the data, verified the coding, and organized the data into themes, as has been previously described (Yoshii et al., 2014). We typed the questionnaire responses, coded anxiety-related raw data by context, and then categorized these codes into subcategory groups. Specifically, we classified 454 codes for maternal anxiety into 66 subcategories. We then extracted subcategories of these anxiety-related factors and further categorized them into category groups. We classified these subcategories into 12 higher-level categories: radiation, child’s physical and mental growth/development, recurrence of earthquakes and tsunamis, financial issues, childrearing environment, living environment, maternal employment, stigma, familial issues, maternal health, childrearing, and the future (Yoshii et al., 2014). This study reports on the categories, subcategories, and coding within the radiation category group.

## 3. Results

### 3.1 Participant Characteristics

The mean age ± standard deviation of participants was 33.0 ± 4.8 years. The most common number of children was two (110 mothers, 42.5%), followed by one child (99, 38.2%). Fourteen participants were currently pregnant (5.4%). Among the participants, 107 were employed (41.3%), and most of these participants (57, 53.3%) worked full-time. Husbands were the most common category of individuals who helped the participants with childrearing (203), followed by the women’s mothers (166). The largest number of participants consulted their husbands when problems arose (201), followed by their own mothers (187).

### 3.2 Anxiety over Radioactivity

In total, 68 codes have been extracted for anxiety over radioactivity. These codes were classified into 18 subcategories. In addition, subcategories were classified into 7 categories. Table 1 describes the category of radiation.

**Table 1:** Mothers’ anxiety over radioactivity

Category	Subcategory	Code
	Effects of radiation on children	Is it okay to have my children eat fish and mushrooms? The volume of radiation contained in children’s food and beverages. What to do about children’s food when considering radiation. I’m worried that I may have had my children eat poison. Are there really no ill effects if radiation accumulates inside children’s small bodies?
	
Food safety	Safety of foodstuffs	Especially food grown in the neighborhood. Radiation effects on food. I cook various vegetables, and I’m worried that we eat food contaminated by radiation, however small its quantity. About water and food safety. For example, what we eat. I buy food at a supermarket now, and I’m worried if it is really harmless to our health. Especially fish and water. That we have to be careful about everything (food, water, etc.) in order to minimize exposure. I buy meat imported from Australia, for example, but I can’t get imported fish. Safety of food because there was an explosion at the nuclear power plant (in Fukushima). Since then, we have had to be careful about food in everyday life.
	
Safety of the Outdoors	Unreliable information	Unreliable information. Is Ishinomaki all right? How soon can we eat produce from the sea and rivers and food in general? What should we do if we have consumed radiation? What foodstuffs to choose. How much homegrown food can be served to my family?
	
	Effects of radiation on children	About radiation effects on children playing outside. For several days just after the disaster when the radiation was in the air, my children were playing outside all day long. From now on, I’ll worry about their health. How to deal with radiation effects on children. Radiation effects. Can we let our children play outside? Radiation from Fukushima Nuclear Power Plant. These days I often go for walks with my children. Whenever they touch the ground or play with things in parks, anything outdoors, I feel nervous. I’m worried when radiation values are high.
	
Effects of radiation on embryos during pregnancy	Effects of radiation	Effects of radioactive substances. I can’t leave Miyagi Prefecture on account of work, but I wonder if I should move to a safe place, including changing my job. About radiation pollution, environmental pollution near public facilities and people’s residences, and future health hazards. About radiation. Since then, we have had to be careful about where to go in everyday life.

	Effects of radiation during pregnancy	Are effects of radiation harmless? I was pregnant last March. Did radiation affect the baby in my womb then? Isn’t a checkup necessary? I’m worried about radiation exposure, because I lived in Miyagi Prefecture while I was pregnant and when I gave birth. I’m worried that not only pregnant women, babies, and children in Fukushima, but also those in neighboring prefectures may well incur health hazards several years from now.

Effects on children	Health of children.	Taking the long view, I’m worried about the future health of our children. Whether health hazards from radiation pollution appear when a person grows to be an adult. Radiation issues. I’m worried if they might influence children’s growth and health in the future. I’m worried how much radiation after the nuclear power plant disaster might affect children’s growth. Radiation. I’m really nervous. Every day the idea of what I should do if my child gets sick in the future eats away at me, and it’s driving me crazy. I can’t work it out in any way. I have no money to move my child to a safe place, and it hurts me, too. I heard about a friend of mine whose child has 200 B/q of internal radiation exposure. I wonder about the children in Miyagi.

	The children’s future	The children’s future from now on. I live within 100 km from the Nuclear Plant (Fukushima). Is it all right to continue living here? I can’t put my worries to rest when I think of a future in which something can happen to my children. More about nuclear power plants than about earthquakes. Is the future of our children all right?

	Effects of radiation on embryos during pregnancy	I want to have a baby, but does radiation have any effect on an embryo? I’m most worried about radiation including damage caused by harmful rumors, whether I raise a child or not.

	Effects of radiation pollution on children	I’m anxious about radiation effects, because I’m in the middle of raising children. About having our children exposed to radiation pollution because their parents have decided to stay here. I wasn’t so worried before, but now I’m anxious about how soon radiation effects may appear in children. Effects of radioactive substances emitted from the Fukushima Daiichi Accident on children.

Radiation effects	Radiation issues	Radiation issues. I live within 80 km from the power plant. Radiation. Effects of radioactive substances. Nuclear power plant and its effects. Worried about radiation near the power plant. Radiation pollution. Worried about how the radiation effects appear. That worries over radiation pollution have not yet disappeared.

	Cesium issues	Cesium issues.

Economy	Economic effects	I’m a farmer and I grow flowers and rice. I’m worried that it might be impossible to sell them because of concerns over radiation.

Distrustful feelings about announcements	Distrustful feelings about safety of foods	I’m worried whether the vegetables we now have our children eat are really safe. I think foods sold at stores are inspected, but I wonder if I can trust them because they are served to children.

	Distrustful feelings about radiation values	How high was the radiation in Sendai at the time of the disaster? I’m anxious to know because it has not been disclosed at all. valign="middle"How well can we trust radiation values? I’m worried there will be damage to children’s health.bI’m very much worried about radiation effects. The Government’s standards and measures are totally untrustworthy. I don’t think they are enough to ensure health.

	Distrustful feelings about Secrets	I would like everything to be made public without hiding anything. Aren’t they hiding the fact that it is in fact dangerous?

	Distrustful feelings about safe	We can’t believe it when we are told it’s safe. I can’t believe it is safe, like playing outside. Aren’t they hiding the fact that it is in fact dangerous? Nuclear power plant issues. Please establish what we should do to avoid harm to our bodies. We have no idea right now.

	Distrustful feelings about media	I can’t trust what the media says, however much they say don’t worry.

## 4. Discussion

This study demonstrated that mothers raising their babies and infants in Miyagi Prefecture were anxious about radiation and its possible effects on diet, play, and growth in children. Based on this finding, we summarized and reviewed the measures taken by relevant organizations and bodies, as presented below.

### 4.1 Lifesaving Water

On March 23, 2011, 12 days after the disaster, the Tokyo Metropolitan Government Bureau of Waterworks recommended that Tokyo residents refrain from using tap water to prepare milk for babies. It announced that Tokyo tap water contained 210 Bq/kg of radioactive substances, which was higher than the standard value. The next day, the Japan Radiological Society responded to this announcement by publishing a web article titled, “To pregnant women and families with children—a reminder about the health hazards of tap water” (Japan Radiological Society HP). The following statements are excerpts from this article.

- Governments consistently monitor the level of radioactive substances contained in tap water. The Bureau’s announcement is to call attention to temporarily higher than usual values.

- If a pregnant woman drinks tap water containing this level of radioactive substances, there will be no effects on the baby in the womb. Do not worry, and act as before.

- Even if she drinks tap water that contains a small amount of radioactive substances as in the present situation, even less than that amount will get into the mother’s milk, so there is no need to search for other sources of water.

- The standard value mentioned here concerns water used to prepare babies’ milk. This is a very strict standard. Even if tap water contains more radiation than this value, you do not have to use other water to cook your children’s food. Tap water will have no ill effects on your children’s health.

However, the Japan Society of Obstetrics and Gynecology published the following comment on its website (Japan Society of Obstetrics and Gynecology HP).

“At present, it is estimated that if pregnant or lactating women drink lightly polluted tap water every day, no health damage will be inflicted on the mother or her baby/embryo. Also, no damage to the health of babies and infants is thought to occur if mothers continue breastfeeding. However, less exposure is safer because embryos, babies, and infants are more vulnerable compared with adults. Therefore, when alternative water other than lightly polluted tap water is available, it is advised to use it.”

Furthermore, the Governor of Miyagi Prefecture announced data on tap water in the prefecture where the study subjects resided and confirmed its safety on March 25, 2011. “Radioiodine was detected at a level of 10 Bq/kg in Shiroishi City, 5 Bq/kg in Kami-machi, and 4 Bq/kg in Taiwa-cho. These are all well below the standard value. Radioactive cesium was not detected at all.”

**Figure 1:**
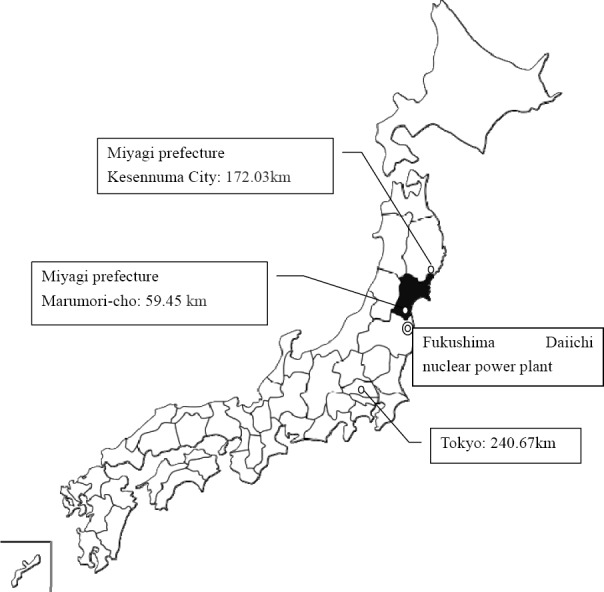
Distance from Fukushima Daiichi Nuclear Power Plant

### 4.2 Food for Nourishment

One year after the nuclear power plant accident in Fukushima, the Japanese government set a new standard for radioactive materials in food (Yamamoto et al., 2012). As an emergency measure, it lowered the yearly limit for maximum radiation exposure through food from 5 mSv (food intake restriction index suggested by the Nuclear Safety Commission based on the Food Sanitation Act) to 1 mSv (Yamamoto et al., 2012). The maximum limit for all food was 100 Bq/kg, not including drinking water, green tea, milk, or baby food intake. The maximum limit for drinking water was 10 Bq/kg and 50 Bq/kg for milk and baby food. On March 25, 2011, in Miyagi Prefecture (where the survey subjects resided), measurements began to be taken of the levels of radioactive material contained in agricultural, forest, and fishery products produced in the prefecture. Findings were as follows (Miyagi Prefectural Government HP).

#### 4.2.1 Rice

Rice is a staple food in Japan that is harvested once a year, around September and October. In September 2011, every municipality in Miyagi Prefecture inspected radioactive cesium levels in rice and confirmed the safety of this food:

Density of radioactive cesium (Bq/kg)

Cs-134: minimum value was lower than 20 Bq/kg, maximum value 48.5 Bq/kg

Cs-137: minimum value was lower than 20 Bq/kg, maximum value 53.1 Bq/kg

#### 4.2.2 Fish

The eastern part of Miyagi Prefecture faces the Pacific Ocean and is famous for rich sea products. However, in May 2012, following the nuclear plant accident, the Prime Minister ordered the restriction of shipments of Japanese sea perch, Pacific cod, and flounder caught off the coast of Miyagi, based on Article 20, Paragraph 3 of the Act on Special Measures Concerning Nuclear Emergency Preparedness. This was done because radioactive cesium (>100 Bq/kg) was detected in these fish.

#### 4.2.3 Vegetables

The March 28, 2011 announcement on vegetables grown in Miyagi Prefecture stated that radioiodine was determined to be well below the standard: 293.8 Bq/kg in spinach in Kawasaki, 623.9 Bq/kg in edible chrysanthemum in Watari, and 372.6 Bq/kg in Japanese mustard spinach in Sendai. The test results for radioactive cesium were similar: 4.6 Bq/kg in spinach in Kawasaki, 5.8 Bq/kg in edible chrysanthemum in Watari, and 119.8 Bq/kg in Japanese mustard spinach in Sendai. Therefore, the announcement confirmed that agricultural products were safe and that they would not adversely affect health. However, later, in January 2012, a shipment restriction was issued for shiitake mushrooms grown on beds or logs and for edible wild plants.

#### 4.2.4 Breast Milk

On April 20, 2011, iodine-131 was found in breast milk from a mother living in Fukushima Prefecture, where the Fukushima Daiichi Nuclear Power Plant is located. Afterwards, in late May, radiation in breast milk was tested among mothers residing in Yamagata, Miyagi, Fukushima, Ibaraki, Tochigi, Gunma, and Kochi prefectures. A very small amount of cesium was detected only in the milk from mothers living in Fukushima. Nothing was detected in the breast milk from mothers living in Miyagi (Kubo et al., 2012).

#### 4.2.5 Comment from the Japan Society of Obstetrics and Gynecology

On July 21, 2011, the Society published a comment on its website titled, “Advice to pregnant and lactating women who are worried about radioiodine contained in foodstuffs” (Japan Society of Obstetrics and Gynecology HP). The comment included an example of a radiation value after eating a steak containing radioiodine, and then explained that no one would suffer adverse health effects unless they were exposed to extremely high amounts of radiation from the air or foodstuffs. It then pointed out that people should try to reduce their total level of exposure by, for example, avoiding highly polluted food.

### 4.3 Playgrounds That Promote Children’s Growth

#### 4.3.1 Miyagi Prefecture

Measurement of air radiation levels in Miyagi Prefecture began on March 15, 2011. Average levels from 17:00 to 24:00 on March 15, 2011 in Sendai City were 0.158 μGy/h (minimum, 0.083 μGy/h; maximum, 0.199 μGy/h). Before March 11, 2011, usual readings measured at monitoring posts were 0.0176–0.0513. As of June 3, 2012, one year and three months after the disaster, levels were 0.056 μSv/h [read at 9–10 a.m. by a monitoring post (post height was 9.5 m from the ground)] and 0.061 μSv/h (read approximately 1 m from the ground) (Nuclear Regulation Authority HP). Therefore, no directives (e.g., a curfew) were deemed necessary in Miyagi. As of June 11, 2013, decontamination works have been completed at 160 points in all, mainly in parks and schools. These works are ongoing (Radiation Information Site Miyagi).

#### 4.3.2 Excerpts From a Comment by the Nuclear Regulation Authority (Nuclear Regulation Authority HP)

“Since March 17, 2011, the volume of radioactive substances emitted into the air from Fukushima Daiichi Nuclear Plant has consistently decreased. However, we should exercise a degree of caution regarding radioactive substances accumulating on the ground or inside buildings. An evacuation order has been issued for each area in which there is a radiation threat to human health, so do not worry excessively in areas with no such order in place. A possible guideline: The International Commission on Radiological Protection (ICRP) issued a statement on March 21: ‘As a reference level, it is possible to consider a margin of 1 mSv to 20 mSv per year for the general public in this case where emergency conditions have returned to normal.’ If we simply assume our daily cycle includes 8 hours of outdoor activity plus 16 hours of indoor (inside a wooden building) activities and then calculate the level, the reference level will be 3.8 mSv/h outside. If we decrease the time spent outside, total radiation exposure per year will naturally go down.”

“What precautions should be taken at playgrounds that promote children’s growth?

- Take care that mud or sand does not get into your mouth.
- Do not drink water from a river or pool. Drink water that is fit to drink.
- Wash mud or sand away from your hands and face after playing outdoors.
- Brush dust off your clothes before entering the classroom or home.
- It is sufficient to wash your hair as before.
- It is safer to use an umbrella when it is raining.”

### 4.4 Anxiety over Radioactivity Exposure in Mothers

On March 12, 2011, the government issued an evacuation order for people living within a 20-km radius of Fukushima Daiichi Nuclear Power Plant. Disaster-stricken Miyagi Prefecture is located north of Fukushima Prefecture. Marumori at the southernmost point of Miyagi Prefecture is 59.45 km from Fukushima Daiichi Nuclear Power Plant, and Kesennuma at the northern point is 172.03 km away from the plant. No evacuation zones were designated for any area of Miyagi Prefecture. Since the accident, various research institutes have reported that measurements of both external and internal individual exposure doses in residents surrounding the Fukushima Daiichi Nuclear Power Plant were less than the guideline exposure (Orita et al., 2013). Nevertheless, anxiety over radiation exposure was still found to be a major problem among mothers in Miyagi Prefecture. Based on studies assessing the effect of food on health, the Food Safety Commission of Japan concluded that extra cumulative effective doses (i.e., cumulative effective doses of radiation during a lifetime) of roughly >100 mSv could increase the risk of adverse health effects (Kitaike et al., 2012). However, there is an undeniable possibility that the health effects of low-dose radiation have not been identified by epidemiological research considering various factors (Kitaike et al., 2012). This fact may have amplified the mothers’ anxiety. Mothers were worried about radiation in food, and some felt guilty having to feed their children food that might be contaminated. Some preferred foreign meat to domestic products. Some mothers were spending days worrying about radioactive substances at their children’s playgrounds. Mothers’ anxiety might have resulted from the dissemination of conflicting opinions and misleading information by apparent experts in the fields of radiology, radiological protection, radiobiology, and medicine, as well as from insufficient knowledge and understanding about radiation exposure and its health hazards (Ogiso et al., 2012). The accuracy and informative content of public information disseminated to the average citizen in the initial months after the disaster was highly problematic (Alder-Collins et al., 2013). Internet sites and blogs spread the conflicting opinions of scholars, journalists, scientists, the Nobel Peace Award holder Helen Caldicott, and physicians (Alder-Collins et al., 2013). Previous studies following the Chernobyl accident reported carcinogenic and genetic risks that increased with increasing radiation exposure even at low radiation doses (Kakamu et al., 2013). The most critical issue when considering the effects of radiation on children’s health is the increase in thyroid cancer, as clearly demonstrated among people who were children or adolescents at the time of the Chernobyl accident (Fushiki et al., 2013). Busby reported that even very low-dose radiation exposure (cumulative absorbed doses to the fetus of 0.02–2 mSv) increased infant leukemia after Chernobyl (Kakamu et al., 2013). This apparently confusing information contributes to a deep distrust of official communications and is fueling high levels of public anxiety about what may happen in the future (Alder-Collins et al., 2013). The Ministry of Education, Culture, Sports, Science and Technology initially set the standard for radiological dosage at 1–20 mSv per year with an outdoor activity time limit, but soon revised it to less than 1 mSv per year.

Such inconsistency in the government’s response, confusing pieces of public information, and the non-existent risk management of nuclear hazards in Japan are likely to lead mothers to distrust government announcements. In particular, this study has demonstrated that government explanations cannot allay anxiety over radiation by mothers raising babies and infants. The Great East Japan Earthquake was a rare catastrophe that was a combination of two natural disasters (earthquake and tsunami) with a man-made disaster (radiation accident). Even now, almost three years later, reconstruction has not progressed as expected. At Fukushima Daiichi Nuclear Power Plant, a newly discovered problem of polluted water leakage has become more serious and is deserving of international attention. Scholars and scientists must take the findings that mothers still have anxiety over radiation seriously and commit to solving problems and confront them head-on. In this pursuit, we both want and need the continuing concern and support from epidemiologists worldwide.

## 5. Conclusion

This study confirmed that concrete types of anxiety over radiation were keenly felt by childrearing mothers who have experienced the disaster. Our findings suggest the need to provide accurate information to these mothers who are otherwise inundated with confusing and varied information while trying to raise their children.
